# Evaluating quality of care for patients with rotator cuff disorders

**DOI:** 10.1186/s12913-018-3375-4

**Published:** 2018-07-20

**Authors:** Breda H.F. Eubank, Mark R. Lafave, J. Preston Wiley, David M. Sheps, Aaron J. Bois, Nicholas G. Mohtadi

**Affiliations:** 1 Department of Health and Physical Education, Faculty of Health, Community, and Education, Mount Royal University, 4825 Mount Royal Gate SW, Calgary, Alberta T3E 6K6 Canada; 2 Sport Medicine Centre, Faculty of Kinesiology, University of Calgary, 2500 University Drive NW, Calgary, Alberta T2N 1N4 Canada; 3 Division of Orthopaedics, Department of Surgery, University of Alberta, 116 St & 85 Ave, Edmonton, Alberta T6G 2R3 Canada; 4 Division of Shoulder and Elbow Surgery, Section of Orthopaedic Surgery, Cumming School of Medicine, University of Calgary, 2500 University Drive NW, Calgary, Alberta T2N 1N4 Canada; 5 Sport Medicine Centre , Faculty of Kinesiology, University of Calgary, 2500 University Drive NW, Calgary, Alberta T2N 1N4 Canada

**Keywords:** Healthcare services, Survey, Quality of healthcare, Rotator cuff disease, Patient satisfaction, Waiting times, Resource utilization

## Abstract

**Background:**

Measuring quality in healthcare is vital in evaluating patient outcomes and system performance. The availability of reliable and valid information about the quality of care for patients presenting with rotator cuff disorders (RCD) in Alberta, Canada is scarce. The objective of this study is to measure quality of care for patients with RCD in order to identify areas of improvement.

**Methods:**

This study employs descriptive survey research design. Between March 2015 and November 2016, a convenience sample of patients presenting with chronic, full-thickness rotator cuff tears to two sport medicine centres in Calgary and Edmonton, Alberta completed two questionnaires: the Healthcare Access and Patient Satisfaction Questionnaire (HAPSQ) and the Rotator Cuff Quality-of-Life Index (RC-QOL). Data collected using both questionnaires were used to make judgments about quality of care. Quality of care was evaluated using six dimensions of quality defined by the Alberta Quality Matrix for Health: accessibility, acceptability, efficiency, effectiveness, appropriateness, and safety. Data was also used to compare current patient clinical pathways to ideal clinical pathway algorithms and used to make judgments about the appropriateness and safety of healthcare practices.

**Results:**

One hundred seventy-one patients participated in the study. The longest mean waiting times for medical services in Alberta were for magnetic resonance imaging (MRI) received in the public sector (103 days) and consultation by orthopaedic surgeon (172 days). Patient satisfaction with respect to quality of care was lowest for emergency room physician and highest for orthopaedic surgeon visits. Patients were treated by a mean of 2.5 physicians (SD: 0.77, range: 2–7). The total aggregate average cost per patient was $4541.19. The mean RC-QOL score for all patients was 42 (SD: 22). Only 54 patients (64%) requiring surgery were able to consult with a surgeon within benchmark timeframes. A comparison of current to ideal clinical pathway algorithms found that 38 patients (22%) experienced indirect clinical pathways, whereby care was fragmented and patients received care from multiple and often, redundant healthcare professionals.

**Conclusion:**

There is a discrepancy between current and ideal clinical pathways whereby some patients are experiencing quality of care that is inefficient, disjointed, and less than ideal.

## Background

Measuring quality in healthcare is vital in evaluating patient outcomes and system performance. The Health Quality Council of Alberta has created a framework, the Alberta Quality Matrix for Health (AQMH), that can be used to measure quality in healthcare [1]. This framework consists of six dimensions of quality that measure patient experience and system performance. Quality of care is defined by a patient’s experience in the health system [2]. High quality care (i.e.*,* ideal clinical pathways) occurs when a patient comes into contact with the health system and the system is seen as accessible, acceptable, efficient, effective, appropriate, and safe.

Rotator cuff disease ranks among the most prevalent of musculoskeletal (MSK) conditions [3–6]. Management of this disease is complex, and a multitude of treatment options exist for patients [7]. Like other orthopaedic problems that are managed within the public sector, patients experience lengthy waiting times; variations in quality and access to care; inefficient use of healthcare resources; lack of coordination between different disciplines and professional specializations; and physicians that are inadequately trained to manage MSK complaints [8–11]. Currently, there is no consistent or comparable set of data to determine the impact of many health services across the country [12]. There exists only inaccurate data that is either difficult to access or non-existent [13].

Quality of care must be responsive to clearly identified needs of the population based on the evidence-based information that is available. There is insufficient information available to evaluate the quality of care that patients receive for their rotator cuff disorder (RCD) in Alberta, Canada (Alberta herein). Therefore, the goal of this study is to use the Healthcare Access and Patient Satisfaction Questionnaire (HAPSQ) [14, 15] and the Rotator Cuff Quality-of-Life Index (RC-QOL) [16] to collect information that can be used to inform the current quality of care in Alberta for patients presenting with chronic, full-thickness rotator cuff tears. The availability of reliable and valid data will allow decision-makers to understand the patient population and evaluate the quality and safety of patient care that currently exists in order to make evidence-based and patient-centred decisions.

## Methods

### Design

To evaluate the quality of care for RCD, patients presenting with chronic, full-thickness rotator cuff tears were recruited from the two largest cities in Alberta, Canada between March 2015 and November 2016. The inclusion and exclusion criteria for this study are presented in Table 1. Convenience samples were recruited prospectively from patients referred to five different orthopedic surgeons at either the University of Calgary Sport Medicine Centre in Calgary, Alberta, or the University of Alberta Glen Sather Sports Medicine Clinic in Edmonton, Alberta. Patients presenting with chronic, full-thickness tears are often treated using conservative, non-operative management or surgery [17–20]. Two groups of patients were invited to complete online versions of both the HAPSQ and RC-QOL. Group 1 included patients that did not require immediate surgical management and were treated conservatively with a non-operative rehabilitation program. Patients unable to achieve pain-free status with improved range-of-motion after 6 weeks were provided additional means of pain control [i.e., oral non-steroidal anti-inflammatory drugs (NSAIDs) medication and/or injectable corticosteroids]. Group 2 consisted of surgically treated patients who had confirmed surgical dates or had already received surgical management for their shoulder problem. These two groups of patients were targeted for this study to provide a representative sample of patients currently presenting to the healthcare system. This study employed descriptive survey research design and was approved by the Conjoint Health Research Ethics Board at the University of Calgary.

**Table 1:** Inclusion and exclusion criteria

Inclusion criteria	Exclusion criteria
• Ages ≥ 18 years old • English-speaking and literate • Chronic, full-thickness rotator cuff tear confirmed by ultrasonography or magnetic resonance imaging	• Concomitant symptomatic pathology of the affected shoulder (i.e., instability, osteoarthritis) • Significant cervical spine pathology or radiculopathy • Medical gain issues (i.e., Workers’ Compensation or litigation) • Unable or unwilling to complete study outcomes • Unable or unwilling to provide informed consent

### Outcome measures

According to the Health Quality Council of Alberta, quality of care can be evaluated by six dimensions: accessibility, acceptability, efficiency, effectiveness, appropriateness, and safety [1]. In the current study, quality of care was measured using two patient-reported outcome measures: the Healthcare Access and Patient Satisfaction Questionnaire (HAPSQ) [21] and the Rotator Cuff Quality-of-Life Index (RC-QOL) [16]. Both questionnaires have been found to be reliable and valid within the context of patients presenting with chronic, full-thickness rotator cuff tears to primary, secondary, and tertiary healthcare settings [16, 21, 22]. The HAPSQ is able to only assess five of six AQMH’s quality dimensions: acceptability, accessibility, efficiency, appropriateness, and safety. Therefore, effectiveness was evaluated using the RC-QOL.

The HAPSQ is a self-administered, multipurpose web-based questionnaire that collects information related to healthcare utilization, access, and patient experiences [21]. Individual items are grouped into sections of the questionnaire rather than scales: use of physician services (e.g., general practitioner/family physician, orthopaedic surgeon) (4 items); use of diagnostic investigations (3 items); surgery (2 items); use of complementary allied medical treatments (e.g., physical therapy, massage therapy) (3 items); out-of-pocket expenses (3 items); lost wages (4 items); patient satisfaction rating of care (2 items); patient expectations around acceptable waiting times (1 item); and demographic information (7 items). The HAPSQ has 29 fixed items, however, the total item count within several sections can vary depending on the quantity of services rendered or items purchased. For example, if the patient received care from two family physicians and one surgeon, the number of items in that section would increase to 12 (3 physicians × 4 items). If the patient purchased five out-of-pocket expenses, the number of items would increase to 15 (5 expenses × 3 items). The HAPSQ is a descriptive, health information tool. Therefore, it does not possess one composite score. Instead, items from different scales are combined to provide health information; whereby results can be used to make a judgment about the accessibility, acceptability, efficiency, appropriateness, and safety of care.

The RC-QOL is a patient-reported health instrument that measures quality-of-life [16]. The RC-QOL contains 34 questions and five subscales: symptoms and physical complaints (16 items); work-related concerns (4 items); recreational activities, sports participation, or competition concerns (4 items); lifestyle concerns (5 items); and social and emotional concerns (5 items). The RC-QOL possesses a composite average score out of 100; whereby 0 indicates the worst quality-of-life and 100 indicates the best.

#### Accessibility

Accessible health services are defined by the AQMH as those “obtained in the most suitable setting in a reasonable time and distance” [1]. Accessibility was measured using patient-reported waiting times (i.e., patients provided dates of visits and reported time spent waiting in days, weeks, months, or years). Using standardized definitions, developed by the Western Canada Waiting List Project [23], the following waiting periods were identified: waiting for primary care; waiting for initial surgical consultation; waiting for diagnostic imaging; waiting for surgery; and total waiting time. Waiting period definitions are presented in Table 2.

**Table 2:** Waiting period definitions

Waiting period	Definition
Waiting for ambulatory care	Number of hours spent in the waiting room of a hospital before receiving physician care
Waiting for primary care	Number of days between date of request for an initial assessment and date of the primary care visit
Waiting for initial surgical consultation	Number of days between date of referral and date of consultation by an orthopaedic surgeon
Waiting for diagnostic imaging	Number of days between the requisite date for a test and date of examination
Waiting for surgery	Number of days between decision to treat surgically and date of surgery
Total waiting time (Group 1)	Aggregate number of days spent waiting for primary care to initial surgical consultation
Total waiting time (Group 2)	Aggregate number of days spent waiting for primary care to surgery

Group 1: patients that did not require immediate surgical management and were treated conservatively with a non-operative rehabilitation program. Group 2: patients who had confirmed surgical dates or had already received surgery

#### Acceptability

Acceptable health services are defined by the AQMH as being “respectful and responsive to user needs, preferences, and expectations” [1]. Acceptability was evaluated by asking patients to indicate their level of satisfaction with respect to: 1) time spent waiting for physician consultation or diagnostic services; and 2) quality of physician care received. Responses were assessed on a 100 mm visual analogue scale from 0 “extremely dissatisfied” and 100 “extremely satisfied”.

#### Efficiency

Efficient health services are defined by the AQMH as “resources that are optimally used in achieving desired outcomes” [1]. In this study, efficiency was evaluated by measuring actual healthcare consumption and calculating associated injury-specific costs. The HAPSQ was used to estimate the volume of healthcare supplied and utilized by the patient. Cost estimates were calculated by multiplication of patient-reported utilization volume and unit costs. Unit costs were estimated using standardized costing methods provided by the Canadian Agency for Drugs and Technology in Health (CADTH) in the Guidance Document for the Costing of Healthcare Resources in the Canadian Setting [24]. Cost estimates were divided into costs to the province; costs to private insurance companies, and costs to the patient. Costs to the province were defined as the total costs associated with use of diagnostic investigations, use of physician services, and surgery. Unit costs for the province were obtained from detailed costing data from the Government of Alberta [25, 26], and the Canadian Institute for Health Information [27]. Costs to private insurance companies were defined as costs that were covered by third party insurance such as prescription medicine and complementary allied medical treatments. Unit costs for prescription medicine were obtained from the Government of Alberta’s Interactive Drug Benefit List [28]. Unit costs for complementary allied medical treatments were estimated using billing guidelines and/or fee schedules provided by provincial governing bodies for each profession [29, 30]. If fee schedules were not available, unit costs were estimated using average retail pricing. Costs to the patient were defined as the total cost of out-of-pocket expenses incurred by the patient including lost wages. Lost wages were estimated by multiplying the number of days taken off work by hourly occupational wage estimates from the Alberta Government’s Alberta Listing Information Service website [31]. Variables used to estimate out-of-pocket expenses included over-the-counter medication, healthcare appliances and aids, and private diagnostic services. Unit costs for these variables were estimated using average retail pricing. All cost estimates were calculated in Canadian dollars (CAD$). Cost transformations were not adjusted for annual inflation and deflation factors during the 2015–2016 time horizon.

#### Effectiveness

Effective health services are defined by the AQMH as being “based on scientific knowledge to achieve desired outcomes” [1]. Effectiveness refers to the efficacy of an intervention in providing the best outcome for the patient [1]. Reliable and valid instruments that measure patient-reported health outcomes (i.e., quality-of-life) can be used to measure change within patients in order to quantify the health benefits of such interventions [32]. Patients were asked to complete the RC-QOL to provide baseline quality-of-life scores.

#### Appropriateness

The AQMH defines appropriate health services as being “relevant to user needs and are based on accepted or evidence-based practice” [1]. Appropriateness was evaluated by comparing patient expectations around acceptable waiting times, utilization of healthcare resources, and clinical care pathways to evidence-based benchmarks. First, appropriateness was measured by comparing actual waiting times to patient-reported expected waiting times for diagnostic and physician services. Secondly, patient-reported health resource utilization was compared to ideal standards of care developed using consensus methods by an expert panel [33]. Finally, HAPSQ responses were used to map clinical pathways experienced by each patient. Clinical pathways detail steps in the care delivery process of each patient. Therefore, appropriateness was evaluated by comparing actual clinical pathways to ideal pathways of care previously developed to standardize care for patients with  chronic, full-thickness rotator cuff tears in an effort to assist in clinical decision-making processes, improve outcomes, and reduce costs [33].

#### Safety

The AQMH defines safe health services as being able to “mitigate risks to avoid unintended or harmful results” [1] HAPSQ responses were used to map clinical pathways experienced by each patient. Clinical pathways detail steps in the care delivery process of each patient. Therefore, safety was evaluated by comparing actual clinical pathways to ideal clinical pathway algorithms in order identify unsafe practices for patients in Alberta.

### Statistical analyses

A post-hoc analysis was completed to identify significant differences between the two cities. The chi-squared (χ^2^) test was used to compare sex, ethnicity, income, and working status. The independent t-test was used to compare age, duration of symptoms, waiting time, patient satisfaction, quality-of-life scores, and costs between Calgary and Edmonton. The paired t-test was used to compare suggested and actual waiting times. The analysis of variance test was used to compare waiting times and patient satisfaction between physician groups. A *p*-value of less than 0.05 was considered statistically significant for all analyses. All statistical analyses were performed using SPSS 17.0 software (SPSS Inc., Chicago, IL, USA) [34].

## Results

Two hundred and fifty-two patients provided consent to participate in this study, in which 171 patients (68%) completed both the HAPSQ and the RC-QOL. Fifty-three patients made no attempt to complete either questionnaire (*n* = 13 Calgary patients and *n* = 40 Edmonton patients) and were lost to follow-up. Twenty-eight patients submitted only partially completed questionnaires (*n* = 17 Calgary patients and *n* = 11 Edmonton patients), in which only demographic information (Page 1) was completed. Information from these questionnaires was not analyzed. The resulting response rates for Calgary and Edmonton were 76% (*N* = 126) and 60% (N = 126), respectively. The main reasons for non-participation were time and effort. Patient demographics and clinical characteristics are presented in Table 3. Overall, the study analyzed 84 questionnaires from Group 1 patients and 87 from Group 2 patients. The mean age for the group was 58 years (SD: 10; range 27–78). The patient population was 61% men (*n* = 104), 89% Caucasian (*n* = 152), and 20% retired (*n* = 35). Thirty-three percent (*n* = 56) reported an annual household income over $100,000. The mean duration of symptoms was 3 years (SD: 5.0; range 0.1–34). Calgary and Edmonton patients were statistically similar in sex [χ^2^(1) = 0.14, *p* = 0.71], ethnicity [χ^2^(1) = 7.2, *p* = 0.41], income [χ^2^(1) = 3.9, *p* = 0.79], working status [χ^2^(1) = 0.81, *p* = 0.40], age [*t*(171) = − 0.56, *p* = 0.58], and duration of symptoms [*t*(171) = 0.55, *p* = 0.13].

**Table 3:** Patient demographics and clinical characteristics

Variable	Overall (*N* = 171)	Calgary (*n* = 96)	Edmonton (*n* = 75)
Age, mean (SD) y	58 (10)	57 (10)	58 (9)
Age, range	27–78	27–78	38–75
Male: *n* (%)	104 (61)	59 (62)	44 (59)
Caucasian: *n* (%)	152 (89)	83 (86)	69 (92)
Retired: *n* (%)	35 (20)	22 (23)	13 (17)
RC-QOL Score (0–100), mean (SD)	42 (22)	43 (21)	42 (23)
RC-QOL Score, range	0–95	5–95	0–91
Duration of symptoms: *n* (%)			
< 1 year	66 (38)	42 (43)	24 (32)
1 to 2 years	25 (15)	12 (13)	13 (17)
2 to 5 years	55 (32)	26 (27)	29 (39)
> 5 years	25 (15)	16 (17)	9 (12)
Income: *n* (%)			
< $25,000	10 (6)	6 (6)	4 (5)
$25,000–49,999	12 (7)	6 (6)	6 (8)
$50,000 – 74,999	21 (12)	14 (15)	7 (9)
$75,000 – 99,999	20 (12)	12 (13)	8 (11)
> $100,000	56 (33)	34 (35)	22 (29)
Prefer not to say	52 (30)	24 (25)	28 (38)
Treatment: *n* (%)			
Group 1	84 (49)	51 (53)	33 (44)
Group 2	87 (51)	45 (47)	42 (56)

*SD* standard deviation

Group 1: patients that did not require immediate surgical management and were treated conservatively with a non-operative rehabilitation program. Group 2: patients who had confirmed surgical dates or had already received surgery

### Accessibility

Table 4 provides the mean and median (med) waiting times for all waiting periods.

**Table 4:** Actual, suggested, and ideal waiting times (days)

		Overall (*n* = 171)					Calgary (*n* = 96)			Edmonton (*n* = 75)		
Variable		MI	MS (SD)	MA (SD)	Median	Range	MA (SD)	Median	Range	MA (SD)	Median	Range
Diagnostic imaging												
	X-ray		6 (11)	3 (6)	1	0–30	4 (6)	1	0–30	2 (5)	0	0–23
	Ultrasound		11 (13)	28 (31)	15	0–180	23 (29)	14	0–180	38 (33)	30	0–120
	MRI public		25 (27)	103 (100)	82	2–611	108 (126)	60	2–611	94 (75)	90	7–300
	MRI private		17 (35)	8 (8)	4	0–30	10 (9)	7	2–30	4 (5)	3	0–14
Physician visits												
Primary care	ER		1 (1)^a^	3 (2) ^a^	2	0–8	2 (2)	2	0–6	3 (2)	2	0–8
	GP/Family	0–7	5 (8)	6 (7)	3	0–30	6 (8)	3	0–30	5 (7)	2	0–30
Specialist	Sport Med.	0–14	13 (9)	37 (48)	21	3–180	41 (55)	20	3–180	28 (25)	21	7–90
	Surgeon	42–84	36 (39)	172 (191)	122	7–1430	175 (232)	62	7–1430	169 (119)	153	7–638
Surgery		84		162 (194)	92	10–1280	137 (159)	64	30–730	188 (223)	121	10–1280
Total wait time				264 (248)	183	14–1491	250 (271)	146	14–1491	282 (215)	247	17–1308
Group 1				157 (133)	119	14–700	142 (142)	91	14–700	180(114)	167	17–372
Group 2				370 (287)	317	24–1491	378 (327)	237	31–1491	362(242)	366	24–1308

*MI* mean ideal waiting times represent the recommended time in days from when a patient decides to seek treatment from a general practitioner (GP)/Family physician, or from the date of referral to a sport medicine physician, surgeon, or for surgery (adopted from Eubank et al., 2016). *MS* mean patient-suggested waiting times. *MA* mean patient-reported actual waiting times. *SD* standard deviation. *MRI* magnetic resonance imaging. *ER* emergency room physician. *GP* general practitioner. *Family* family physician. *Sport Med* sport medicine physician

Group 1: patients that did not require immediate surgical management and were treated conservatively with a non-operative rehabilitation program. Group 2: patients who had confirmed surgical dates or had already received surgery

^a^Waiting time measured in hours

The mean waiting time for ambulatory care was 3 h (SD: 2, med: 2, range: 0–8). The mean waiting time for consultation by a surgeon was 172 days (SD: 191, med: 122, range: 7–1430). An analysis of variance showed that this waiting time was significantly different when compared to the other physician groups [F (3, 298) = 65.7, *p* < 0.001)]. Tukey HSD post hoc test for significance indicated that the mean waiting time to see a surgeon was significantly higher when compared to general practitioner/family physician (mean: 6 days, SD: 7, med: 3, range: 0–30) and sport medicine physician (mean: 37 days, SD: 48, med: 21, range: 3–180).

The mean waiting time for diagnostic imaging is also presented in Table 4. An analysis of variance showed that the mean waiting time for magnetic resonance imaging (MRI) received in the public healthcare system (mean: 103 days, SD: 100, med: 82, range: 2–611) was significantly different when compared to x-ray (mean: 3 days, SD: 6, med: 1, range: 0–30), ultrasound (mean: 28 days, SD: 31, med: 15, range: 0–180), and MRI obtained through a private diagnostic clinic (mean: 8 days, SD: 8; med: 4; range: 0–30) [F (3, 349) = 22.2, *p* < 0.001)]. Tukey HSD post hoc test for significance indicated that the mean waiting time for public MRI was significantly higher when compared to all other diagnostic tests.

The total mean waiting time for all patients was 264 days (SD: 248, med: 183, range: 14–1491). Although patients in Edmonton had slightly longer waiting times over Calgary, this number was not found to be significantly different. In fact, a comparison of all mean waiting times did not result in any significant differences between the two cities. There was a significant difference in total mean waiting time for Group 1 patients (mean: 157 days, SD: 133, med: 119, range: 14–700) compared to Group 2 patients (mean: 370 days, SD: 287, med: 317, range: 24–1491); [*t*(171) = − 6.2, *p* < 0.001)].

Of the 96 patients that received care at the University of Calgary Sport Medicine Centre, 21 patients (22%) were not residents of the city of Calgary. Of these, 10 were from rural townships not within areas surrounding Calgary (e.g., Okotoks, Airdrie). Of the 75 patients that received care at the University of Alberta Glen Sather Sports Medicine Clinic, 20 patients (27%) were not residents of the city of Edmonton. Of these, 7 patients were from rural townships not within areas surrounding Edmonton (e.g., St. Alberta, Sherwood Park).

### Acceptability

Patient satisfaction with respect to quality of care and waiting times are presented in Table 5. The mean patient satisfaction with respect to quality of care was lowest for emergency room physicians at 62% (SD: 33) and highest for surgeons at 90% (SD: 21). An analysis of variance demonstrated that patient satisfaction with respect to quality of care provided by a surgeon was significantly different between the other physician groups [F (3, 339) = 12.9, *p* < 0.001). Tukey HSD post hoc test for significance demonstrated that patient satisfaction for surgeons was significantly higher than emergency room physicians (*p* < 0.001) and general practitioners/family physicians (*p* = 0.01). A comparison of patient satisfaction between Calgary and Edmonton with respect to quality of care did not reveal a significant difference.

**Table 5:** Mean patient satisfaction percentiles with respect to quality of care and waiting times

		OVERALL (*n* = 171)		CALGARY (*n* = 96)		EDMONTON (*n* = 75)	
		Quality of Care [%, (SD)]	Waiting Time [%, (SD)]	Quality of Care [%, (SD)]	Waiting Time [%, (SD)]	Quality of Care [%, (SD)]	Waiting Time [%, (SD)]
Diagnostic imaging							
	X-ray		78 (29)		76 (30)		81 (28)
	Ultrasound		57 (33)		59 (32)		51 (38)
	MRI public		46 (37)		45 (35)		47 (39)
	MRI private		74 (37)		65 (43)		93 (11)
Physician provider							
Primary care	ER	62 (33)	54 (37)	67 (27)	55 (30)	59 (36)	53 (42)
	GP/Family	80 (27)	73 (31)	81 (26)	75 (29)	77 (29)	70 (32)
Specialist	Sport Med.	80 (24)	73 (28)	82 (23)	77 (25)	75 (27)	67 (34)
	Surgeon	90 (21)	60 (35)	89 (23)	61 (34)	90 (18)	59 (36)

*SD* standard deviation. *MRI* magnetic resonance imaging. *ER* emergency room physician. *GP* general practitioner. *Family* family physician. *Sport Med* sport medicine physician

The mean patient satisfaction with respect to waiting time for physician consultation was also lowest for emergency room physician at 54% (SD: 37). Both general practitioners/family physicians and sport medicine physicians had a mean of 73% (SD: 31 and SD: 28 respectively). An analysis of variance demonstrated that patient satisfaction with respect to waiting time suggested a significant difference between the physician groups [F (3, 339) = 12.9, *p* = 0.001). Tukey HSD post hoc test for significance demonstrated that the mean patient satisfaction for surgeons (60%, SD: 35) was significantly lower than general practitioners/family physicians (p = 0.01) and sport medicine physicians (*p* < 0.001). No significant differences were found between Calgary and Edmonton.

The mean patient satisfaction with respect to waiting time for diagnostic services was lowest for public MRI (mean: 46%, SD: 37) and highest for x-ray (mean: 78%, SD: 29). An analysis of variance indicated a significant difference between types of diagnostic imaging [F (4, 356) = 19.2, p < 0.001). Tukey HSD post hoc test for significance demonstrated that the mean patient satisfaction for public MRI was significantly lower than x-ray (*p* < 0.001) and private MRI (mean: 74%, SD: 37, *p* = 0.03), but not for ultrasound (mean: 57%, SD: 33, *p* = 0.14).

### Efficiency

Utilization of provincial healthcare services are presented in Table 6. The mean number of physicians seen by patients was 2.50 (SD: 0.77; range: 2–7). Patients in Calgary most frequently sought care from general practitioners/family physicians (mean: 3.70, SD: 4.18); whereas patients in Edmonton most frequently sought care from sport medicine physicians (mean: 4.00, SD: 4.72). Patients in Calgary and Edmonton receiving care for their RCD used approximately an equivalent amount of diagnostic and physician services. The utilization difference between the two cities was statistically significant in Edmonton regarding the use of public MRI (*p* = 0.02).

**Table 6:** Utilization of provincial healthcare services by patients with rotator cuff disorders

Utilization variable	Overall (*n* = 171) Mean (SD)	Calgary (*n* = 96) Mean (SD)	Edmonton (*n* = 75) Mean (SD)	*p* value*	Utilization ratio^a^
X-ray	1.74 (1.50)	1.63 (1.69)	1.90 (1.14)	0.28	0.86
Ultrasound	1.17 (0.92)	1.08 (0.83)	1.36 (1.07)	0.95	0.79
MRI (public)	0.50 (0.59)	0.41 (0.59)	0.61 (0.57)	0.02*	0.67
Emergency room visits	1.13 (0.42)	1.08 (0.52)	1.15 (0.37)	0.67	0.94
GP/Family physician clinical visits	3.52 (3.78)	3.70 (4.18)	3.30 (3.24)	0.55	1.12
Sport medicine physician clinical visits	3.54 (3.28)	3.32 (2.47)	4.00 (4.72)	0.62	0.83
Surgeon clinical visits	2.67 (2.57)	2.51 (2.93)	2.87 (2.06)	0.37	0.87

*MRI* magnetic resonance imaging, *GP* general practitioner

*p-value significant at < 0.05

^a^Utilization ratio = ratio of utilization for Calgary group patients to utilization for Edmonton group patients

Table 7 summarizes the average costs incurred by patients presenting with RCD in Alberta. The total aggregate average cost per patient was $4541.19 (SD: 2953.23). The total aggregate average cost for the Calgary group per patient was $3832.05 (SD: 2284.83) compared to $5448.91 (SD: 3368.47) for the Edmonton group (*p* = 0.001). The Calgary group thus incurred 70% of the total cost of the Edmonton group. No significant cost difference was found with respect to provincial healthcare costs ($2392.86 vs $2794.98) for Calgary and Edmonton, respectively. However, there was a significant difference in costs incurred by the patient ($284.80 vs $528.89) and private insurance companies ($1154.39 vs $2125.04). Specifically, patients in Edmonton purchased more complementary allied medical treatments and rehabilitation appliances (e.g., Therabands, cold packs, exercise equipment).

**Table 7:** Patient, provincial healthcare, and insurance costs for patients with rotator cuff disorders

Cost variable	Overall (*n* = 171) Mean (SD)	Calgary (*n* = 96) Mean (SD)	Edmonton (*n* = 75) Mean (SD)	*p* value*	Cost ratio^a^
Patient	391.86 (642.36)	284.80 (385.93)	528.89 (822.02)	0.02	0.54
Provincial healthcare	2569.22 (1721.58)	2392.86 (1715.65)	2794.98 (1714.08)	0.17	0.86
Private insurance companies	1580.11 (2016.03)	1154.39 (1357.91)	2125.04 (2536.48)	0.003	0.54
Total	4541.19 (2953.23)	3832.05 (2284.83)	5448.91 (3368.47)	0.001	0.70

*p-value significant at < 0.05

^a^Cost ratio = ratio of costs for Calgary group patients to costs for Edmonton group patients

### Effectiveness

The mean RC-QOL score for all patients was 42 (SD: 22). The mean RC-QOL score was 44 (SD: 22) for Group 1 patients and 41 (SD: 21) for Group 2 patients. A comparison of both treatment groups did not find a significant difference [*t*(171) = 1.1, *p* = 0.28)]. The RC-QOL score of patient in Calgary were not found to be significantly different [*t*(171) = 0.11, *p* = 0.91)] than Edmonton.

### Appropriateness

A comparison of actual waiting times, healthcare resource utilization, and clinical care pathways to ideal clinical standards of care with respect to diagnostic imaging was performed. Benchmark waiting times for physician consultation and diagnostic services were suggested by patients and are presented in Table 4. The suggested mean waiting time for emergency room physician was one hour (SD: 1). The suggested mean waiting time for surgical consultation was 36 days (SD: 39). The suggested mean waiting times for emergency room physician [*t*(33) = 4.0, *p* < .001)], sport medicine physician [*t*(30) = 2.8, *p* = 0.01)] and surgeon [*t*(171) = 5.7, *p* < 0.001)] were significantly different when compared with their actual mean waiting times. The suggested mean waiting times for diagnostic services ranged from 6 days (SD: 11) for x-ray to 25 days (SD: 27) for public MRI. The suggested mean waiting times for ultrasound [*t*(117) = 7.3, p < .001)] and public MRI [*t*(77) = 6.5, p = 0.01)] were significantly different when compared with their actual waiting times.

Eubank et al., 2016 recommended that all patients suffering chronic, full-thickness rotator cuff tears require the following standard shoulder x-rays: true anteroposterior, axillary, and trans-scapular lateral views [33]. Sixteen patients (9%) reported that they had not received any x-rays. Additional recommendations included that an ultrasound be used as the most cost-effective investigation for diagnosing rotator cuff pathology, and that MRI only be ordered by a surgeon primarily for surgical planning purposes [33]. Prior to seeing a surgeon, 77 patients (45%) received an MRI in the public system, and 19 patients (11%) paid out-of-pocket for a private MRI. Fifty-six patients (33%) received both an ultrasound and MRI.

### Safety

Clinical pathways were analyzed for each patient and compared to ideal clinical pathway algorithms [33]. In this comparison, 38 patients (22%) experienced indirect clinical pathways in which patient care was fragmented, and patients sought care from too many and often redundant healthcare professionals. Patient-reported waiting times were also compared to benchmark waiting times [33]. For Group 1 patients, the ideal standard of care for non-operative patients is as follows: consultation with an expert trained and confident to assess and diagnose rotator cuff pathology within 2 weeks after the patient decides to seek medical care, followed by prescription of a 12 week home or supervised physical therapy program [33]. Only 65% of Group 1 patients (54/84) had been received a non-operative program prior to being recruited for the study. Of these, only 24 patients (29%) had successfully completed 12 weeks of physical therapy. In this study, only one patient (1%) met the ideal standard of care for non-operative treatment. For Group 2 patients, the ideal waiting time from when the patient enters the primary healthcare system and surgery is between 12 to 22 weeks for an acute rotator cuff tear, and between 30 and 38 weeks for a chronic tear [33]. Only 40 patients (46%) received surgery within the ideal timeframe. Although levels of satisfaction from these patients were low, no medical complications or harmful experiences were reported by patients during the study period.

## Discussion

Quality of care can be evaluated by collecting adequate, reliable, and valid data using patient-reported outcome measures [35]. Real-time information is critical to determining the quality of care a patient receives, and can provide a complete description of the patient’s clinical pathway. Such information can be utilized to evaluate and improve current healthcare system processes [23, 36]. Two instruments were used to collect such information: the HAPSQ and the RC-QOL. This study is the first step towards evaluating quality of care at a provincial level for patients presenting to the healthcare system with chronic, full-thickness tears of the rotator cuff.

### Accessibility

Accessibility was measured using waiting times and distance. Lengthy waiting times to healthcare services and procedures serve as a barrier to care for many Albertans. According to the Government of Alberta’s Wait Time Registry, 95% of patients requiring interventions of the shoulder experienced a mean waiting time of 315 days for surgery [37]. In this study, patients requiring surgery experienced a mean waiting time of 370 days. The largest delay occurred while waiting for consultation with a surgeon. Group 1 patients experienced a mean waiting time of 157 days, while Group 2 patients experienced a mean waiting time of 172 days.

Another contributing factor to unnecessary waiting times occurred for patients waiting for MRI. Patients in this study spent a mean waiting time of 103 days before undergoing an MRI in the public system; however, this is likely an underestimation as the reported wait time from the Alberta Wait Times Reporting Website currently shows an average wait time of 280 days [37]. The ideal standard of care begins with standardized shoulder x-rays [33]. If additional investigations are warranted, an ultrasound should be obtained to assess the status of the rotator cuff [33] Patients in this study spent a mean waiting time of 28 days waiting for ultrasound; an average difference of 75 days. An MRI is unwarranted in most occasions and should ideally be requested by a surgeon for surgical planning [33]. Ultrasound is the cost-effective investigation for defining full and partial-thickness rotator cuff tears, and is comparable to MRI in both sensitivity and specificity [38]. More importantly, surgical treatment of chronic, full-thickness rotator cuff tears is not always necessary. In fact, non-operative treatment using physical therapy protocols have been previously demonstrated as an effective treatment for chronic rotator cuff tears [19]. It is crucial that primary care physicians and complementary allied medical providers managing patients with chronic, full-thickness rotator cuff tears recognize that a trial of non-operative treatment should be started at the time of the initial clinical presentation, and that MRI and referral to a surgeon be reserved for ‘non-responders’ to the initial line of treatment [33]. Surgery is an invasive procedure and is not always the best option for patients. Prescription and adherence to an early non-operative program can result in successful treatment of chronic, full-thickness rotator cuff tears and serve as an alternative to surgery [17–20], which can reduce utilization of healthcare resources, reduce inappropriate surgical referrals, and save costs to both the healthcare system and the patient.

The services available to rural populations are often very different compared to those available in urban areas [39]. Specifically, access to specialist health service providers and surgical procedures are often restricted to larger medical centres and hospitals found in cities such as Calgary and Edmonton. As such, 17 patients (10%) travelled from rural areas to seek care from specialists.

### Acceptability

Healthcare systems have recently sought to not only achieve a balance in clinical effective and evidence-based care, but also provide services which are judged by patients as acceptable and beneficial [40]. In this study, patient satisfaction with respect to the quality of care received and the length of time spent waiting for physician care were used to evaluate acceptability. Overall, patients were not satisfied with either the quality of care or waiting time of emergency room physicians. In contrast, patients were satisfied with both the quality of care and waiting time of general practitioners/family physicians and sport medicine physicians. Although patients were satisfied with the quality of care received from surgeons, they were unsatisfied with the time spent waiting for care. Patients were the least satisfied when asked about waiting for MRI in the public system.

### Efficiency

Efficiency was evaluated through healthcare utilization and its associated costs. To our knowledge, this is the first study to have measured the direct costs of chronic, full-thickness rotator cuff tears in patients in Alberta. The cost of chronic rotator cuff tears to society is multi-dimensional. Costs to the province made up more than half of the total aggregate costs. The two main cost drivers were diagnostic imaging and physician visits. An ultrasound is the cost-effective investigation for diagnosing rotator cuff pathology [33]. In Alberta, an ultrasound costs approximately $160 [26], whereas a MRI costs approximately $530 [25]; a difference of $370. In this study, 117 patients received an ultrasound; 159 patients received MRI; and 56 patients received both an ultrasound and MRI. This resulted in provincial spending of $113,950.00 for MRI costs compared to $27,680 for ultrasound. This study also demonstrated that patients sought care from an average of two or more physicians before receiving adequate treatment for their problem, with one patient having received care from seven different physicians. Most patients received care from multiple physicians over numerous visits, similar to another study that also found the current state of care to be plagued with an overuse of too many practitioners at the primary care level [14]. Since physician visits are publicly funded in Alberta, healthcare expenditures will continue to rise with the overuse of healthcare resources. Currently, Alberta spends more on physician services than most other provinces and territories in Canada [41].

### Effectiveness

Effectiveness was measured using the RC-QOL to obtain baseline quality-of-life scores as a quantitative measure of the current state of care for patients with chronic, full-thickness rotator cuff tears. Such information can be used to assess the effectiveness of alternative clinical pathways in future studies. As part of this study, baseline quality-of-life scores were obtained for both groups of patients. Mean baseline quality-of-life scores were similarly low at 44 (SD: 22) for Group 1 patients and 41 (SD: 21) for Group 2 patients.

### Appropriateness

Appropriateness of the healthcare system must be measured relevant to user needs. According to this study, patient needs were not met with respect to waiting time. Patients expected significantly lower waiting times from sport medicine physicians and surgeons. Patients also expected significantly lower waiting times for ultrasound and public MRI. With respect to utilization of healthcare resources, MRI and physician services were over-utilized.

### Safety

Patient safety is the cornerstone of high quality healthcare [42]. This corresponds to receiving treatment from the appropriate healthcare providers within the right timeframe to achieve optimal clinical outcomes. Ideally, the most direct clinical pathway should be the most appropriate and safest pathway. The most direct clinical pathway begins when a patient decides to seek medical care for their shoulder complaint and enters the primary healthcare system [33]. Usually they seek care from an emergency room physician or a general practitioner/family physician. If the primary care physician does not feel confident in their clinical assessment skills, they should refer the patient to an expert (e.g., sport medicine physician or non-physician expert) [15]. At this time, it is recommended that all patients with chronic, full-thickness rotator cuff tears be prescribed a 12 week non-operative, physical therapy program (home or supervised) [33]. Patients unable to achieve pain-free status with improved range-of-motion after 6 weeks should be provided additional means of pain control (i.e., oral NSAID medication and/or injectable corticosteroids) [33]. If the patient fails non-operative treatment, they should be referred to a surgeon. If the surgeon and patient collectively decide that surgery is the best option, the patient should receive surgery. Immediate operative repair within 3 months from the onset of symptoms has been proposed to result in better post-operative patient outcomes, earlier return-to-work, and decreased costs [43]. In this study, 38 patients (22%) experienced care that was inconsistent with ideal clinical pathway algorithms. Furthermore, only 40 patients (46%) received surgery within the ideal timeframe. Similarly, studies have shown that conservative treatment of chronic, full-thickness rotator cuff tears results in better outcomes if treatment is started within 6 months of the onset of symptoms [44]. Only one patient (1%) met the standard of care for non-operative treatment. Although no complications were reported in this study, only 24% of all patients received care within appropriate benchmark timeframes.

### Implications

RCD can be long-lasting, debilitating, and costly. As Alberta’s population continues to age, the prevalence of these conditions will increase, thus placing a large economic burden on the already strained healthcare system [45]. The results of this study suggest that the current state of healthcare delivery is fragmented through a complex system, whereby patients are seeking medical care from different physician providers. This presents challenges in providing appropriate care and coordinating access for patients with chronic, full-thickness rotator cuff tears. Ideal clinical pathway algorithms, along with waiting time benchmarks that detail stepwise care for patients throughout primary, secondary, and tertiary healthcare settings, were recently published [33]. The objective of creating such pathways was to ensure that patients were safely and appropriately managed within acceptable timeframes without wasting healthcare resources and worsening health outcomes. Adherence to clinical pathway algorithms will help in decision-making processes and improve patient care.

Recently, there has been a surge in the development of clinical pathways in Alberta focusing on addiction and mental health, cancer care, cardiovascular health and stroke, critical care obesity, diabetes, digestive health, emergency medicine, renal health, respiratory health, seniors’ health, and safer surgical procedures [46]. Although bone and joint health has been designated another priority area, no projects on RCD have been studied. This is the first study to evaluate the current state of healthcare for patients with chronic, full-thickness rotator cuff tears presenting to primary, secondary, and tertiary healthcare settings in Alberta. This study generated important knowledge about the quality of care patients received and identified areas in need of improvement. The findings are similar to those previously studied with respect to patients presenting with acute knee injuries; which found the current state of care to be plagued with lengthy waiting times, unsatisfied patients, bottlenecks for specialist services, inappropriate use of MRI, and a clinical care pathway that utilizes too many medical service providers [14, 15].

### Limitations

This study is not without limitations. The use of convenience sampling may limit the extent to which the results can be generalized to the population of interest; however, patients were recruited from the two largest academic centers in Alberta in an attempt to obtain a representative sample of the population. Overall, the results of this study show similar trends in the quality of care when compared to other reports published across Canada [41, 47].

Another limitation involved sampling bias as all patients in this study were recruited from sport medicine clinics and seen by an orthopedic surgeon. Therefore, information for patients with chronic, full-thickness rotator cuff tears that presented to other physician provider groups or complementary allied medical providers was not captured in this study. The fact that all patients in this study were seen by a surgeon may have also led to response bias and potentially impacted patient satisfaction with respect to the quality of care provided by a surgeon, which was significantly higher than the other physician provider groups. This needs to be further studied given the limited number of surgeons used in this study.

Another sampling bias occurred with patient inclusion criteria. The spectrum of RCD is broad, however, the sample population for the study was limited to patients presenting with chronic, full-thickness rotator cuff tears. This clinical presentation was chosen because anecdotal evidence had suggested this sample of patients to experience difficulty accessing care. It was also selected because an ideal clinical pathway algorithm had previously been developed for this clinical presentation, which allowed the comparison of the current state to the ideal. Therefore, the results presented in this study may not be representative of patients presenting with other RCD such as partial-thickness tears or acute, traumatic tears of the rotator cuff.

Furthermore, it is important to highlight the difference in response rates between Calgary and Edmonton (76 and 60% respectfully), which has the potential to introduce non-response bias to the study. This occurred because more Calgary patients received completion requests in person during subsequent follow-up visits; whereas more Edmonton patients were followed-up over email. This finding is consistent with previous studies that found face-to-face requests to be more successful than email requests [48]. A future sensitivity analysis could be performed to further examine the impact of this difference; however, bias resulting from non-response in surveys is difficult to assess since information about non-responders is rarely available. It is important to note that non-response bias in this study would have been greater had the response rates been lower. The response rates, however, were moderately high compared to those published in the literature, which suggest benchmark response rates of 35 to 50% [49]. Nevertheless, this may reflect further concerns regarding the generalizability of the information.

Finally, patient recall was used to create a history of care throughout the patients’ continuum of care. Although there is some skepticism about the reliability and validity of self-report and patient recall, many studies have found patient recall of healthcare events to be reliable [50, 51]. The HAPSQ and the RC-QOL permitted patients to provide information that was tangible and unique. Currently, this presents an inexpensive and efficient method for communicating both clinical and cost information.

## Conclusion

This study demonstrated a worthwhile and useful approach to evaluating the current quality of care for patients with chronic, full-thickness rotator cuff tears in Alberta, Canada. The HAPSQ is a self-report measure of waiting time, patient satisfaction, healthcare utilization, and cost that has demonstrated reliability and validity. Together with the RC-QOL, the six dimensions of quality were measured and evaluated. The results of the study demonstrated that healthcare accessibility was hindered by longer than expected waiting times. Acceptability (i.e., patient satisfaction) varied depending on physician type and waiting times. Inefficiencies in cost and healthcare resource utilization were identified. Effectiveness could not be measured in this study, however, quality-of-life scores were obtained to provide a baseline quantitative measure for a future study. The current quality of care for patients presenting with chronic, full-thickness rotator cuff tears is inappropriate, as patient expectations were not met, and patients experienced clinical pathways that were inefficient, disjointed, and often redundant. Although no harmful or adverse reactions were reported by patients as a result of their experiences, thus by definition considered “safe”, the majority of patients failed to receive treatment from appropriate healthcare providers within the right timeframe. Consequently, much work is required to improve the level of care for patients with chronic, full-thickness rotator cuff tears. Measuring quality of care, however, is a necessary first step towards an effort to address the challenges that currently exist.

## Abbreviations

AQMH: Alberta Quality Matrix for Health
CAD$: Canadian dollars
HAPSQ: Healthcare Access and Patient Satisfaction Questionnaire
ICC: Intraclass correlation coefficient
Med: Median
MRI: Magnetic resonance imaging
MSK: Musculoskeletal
NSAIDs: Non-steroidal anti-inflammatory drugs
RCD: Rotator cuff disorders
SD: Standard deviation
χ2: Chi-squared
